# ANTIBIOTIC PROPHYLAXIS IN LAPAROSCOPIC CHOLECISTECTOMY: IS IT WORTH DOING?

**DOI:** 10.1590/0102-6720201600030010

**Published:** 2016

**Authors:** Márcio Alexandre Terra PASSOS, Pedro Eder PORTARI-FILHO

**Affiliations:** 1 University Hospital Severino Sombra, Vassouras, RJ, Brazil; 2 Gafréé Guinle Hospital - UNIRIO, Rio de Janeiro, RJ, Brazil

**Keywords:** Laparoscopic cholecystectomy, Antibiotic prophylaxis, Surgical infection.

## Abstract

**Background:**

Elective laparoscopic cholecystectomy has very low risk for infectious complications, ranging the infection rate from 0.4% to 1.1%. Many surgeons still use routine antibiotic prophylaxis

**Aim:**

Evaluate the real impact of antibiotic prophylaxis in elective laparoscopic cholecystectomies in low risk patients.

**Method:**

Prospective, randomized and double-blind study. Were evaluated 100 patients that underwent elective laparoscopic cholecystectomy divided in two groups: group A (n=50), patients that received prophylaxis using intravenous Cephazolin (2 g) during anesthetic induction and group B (n=50), patients that didn't receive any antibiotic prophylaxis. The outcome evaluated were infeccious complications at surgical site. The patients were reviewed seven and 30 days after surgery.

**Results:**

There was incidence of 2% in infection complications in group A and 2% in group B. There was no statistical significant difference of infectious complications (p=0,05) between the groups. The groups were homogeneous and comparable.

**Conclusion:**

The use of the antibiotic prophylaxis in laparoscopic cholecystectomy in low risk patients doesn't provide any significant benefit in the decrease of surgical wound infection.

## RESUMO

**Racional:**

A colecistectomia laparoscópica eletiva apresenta risco muito baixo para complicações infecciosas, com média de infecção entre 0,4% a 1,1%. Muitos cirurgiões ainda utilizam de rotina profilaxia antibiótica

**Objetivo:**

Avaliar a real necessidade de profilaxia antibiótica em colecistectomias laparoscópicas eletivas em pacientes de baixo risco para infecção do sítio cirúrgico.

**Método:**

Estudo prospectivo, randomizado e duplo-cego, em pacientes submetidos à colecistectomia laparoscópica eletiva, envolvendo 100 pacientes em dois grupos: A (n=50), que receberam profilaxia com cefazolina 2 g intravenoso na indução anestésica; B (n=50), não foi utilizado antibiótico. O desfecho avaliado foi presença de complicações infecciosas de sítio cirúrgico. Os pacientes foram revisados em sete e 30 dias no pós-operatório.

**Resultados:**

As taxas de complicações infecciosas foi de 2% no grupo A e de também 2% no grupo B. Não houve diferença estatisticamente significativa (p>0,05) entre os grupos que foram homogêneos e comparáveis.

**Conclusão:**

A antibioticoprofilaxia na colecistectomia laparoscópica em pacientes de baixo risco não apresenta nenhum benefício significativo na redução da incidência de infecção do sítio cirúrgico.

## INTRODUCTION

Laparoscopic cholecystectomy is widespread and described in the medical literature for the first time in March 1987 by Mouret^15^ in Lyon, France, and later improved by Dubois^6^. Currently it is the "gold standard" surgical procedure for choleystectomy^14^ ^,^ ^25^. Electively it has very low risk for infectious complications, having average infection rate between 0.4-1.1%^1^ ^,^ ^2^ ^,^ ^14^ ^,1719,^ ^23^ ^,^ ^24^, and when compared to laparotomy has a lower incidence of infectious complications^9^ ^,^ ^24^ ^,^ ^25^; however, many surgeons still use it routinely^3^ ^,^ ^11^ ^,^ ^20^ ^,^ ^21^.

The Center for Disease Control in the United States, indicate first generation cephalosporin (cefazolin and cephalothin), when necessary their use for prophylaxis on the biliary surgery^25^.

A large number of studies in the literature points to the non-use of antibiotic prophylaxis in laparoscopic cholecystectomy^18^ ^,^ ^25^; however, some authors have conflicting results endorsing its prophylactic use^12^ ^,^ ^13^; also, there is an increase in the operation cost using it^7^. Thus, there is controversy about the prophylactic routine, especially in low-risk patients.

Surgical infections constitute a significant proportion of infections in hospitalized patients. Bacteria are found in 90% of surgical incisions, increasing from beginning to end of the surgical procedure^6^ ^,^ ^10^ ^,^ ^14^ ^,^ ^15^. 

The aim of this study was to evaluate the need for antibiotic prophylaxis in elective laparoscopic cholecystectomy in patients at low risk for surgical site infection.

## METHOD

**FIGURE 1:**
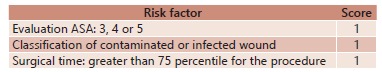
Risk index of surgical site infection by NNIS system

It is a prospective randomized study, double-blind trial of 100 patients with low surgical risk with uncomplicated lithiasic cholecystitis undergoing elective laparoscopic cholecystectomy. It was used NNIS index (National Nosocomial Infections Surveillance, 1991) in order to classify the patients with equivalent risk for postoperative infection in laparoscopic cholecystectomy^14^ ^,^ ^23^ ^,^ ^24^ ^,^ ^25^ ^,^ ^29^. This index establishes the risk of infection of different surgical patients where the risk factors are: a) preoperative assessment score of the American Society of Anesthesiologists (ASA) 3, 4 or 5; b) classification of the operation as contaminated or infected; and c) duration of the procedure. The NNIS index can have values ​​from 0 to 3 (Figure 1) wherein each risk factor is worth 1 point in the score calculation. This classification is simpler to use than others, because the ASA score - it is the variable that measures the intrinsic risk of infection - is much easier to recover from patient charts than the number of diagnoses at hospital discharge and, also can be computed before discharge. The duration of the surgery is indexed to specific surgical procedures, taking into consideration, therefore, the complexity of the procedure. The score NNIS ranges from 0 to 3, with intention to indicate increased risk of infection at the surgical site^5^.

Exclusion criteria were: presence of acute cholecystitis and/or choledocholithiasis; use of antibiotics within 48 h prior to surgery; conversion to laparotomy; patients with renal or hepatic impairment; any state of immunosuppression; regular use or the last 30 days of immunosuppressive drugs; and not signing the consent form.

Patients were allocated through electronic draw, and the results for each patient were placed in sealed envelopes, which were delivered to anesthesiologists responsible for the anesthetic procedure, that ministered or not antibiotics without the surgeon's knowledge.

Patients were divided into two groups: A, received prophylactic cefazolin 2 g intravenously during anesthesia; and B, which received no antibiotic. The operations were performed at the University Gafréé Guinle Hospital - UNIRIO, Rio de Janeiro, RJ, Brazil. The groups were compared for age, gender, comorbidities, surgical time and the calculation of the NNIS score. The outcome were infectious complications on surgical site, ie surgical wound infection and superficial/deep abscesses. The patients were evaluated in seven and 30 days after the operation.

Clinically infectious complications were defined by the typical signs of local or systemic infection as: axillary temperature >37.8º C (excluding the 1^st^ postoperative day), tachycardia, asthenia, accompanied by local pain or purulent collection on surgical site, or signs of inflammation in the wound with no purulent secretion with microbiological confirmation, even without clinical signs of systemic infection^28^. Every discharge from the surgical wound was sent for culture and antibiogram.

### Statistical analysis

The results of the complications and mortality were expressed as percentage. Comparisons between the groups were performed by Chi-square test, implemented by SPSS 20. Distributions of continuous variables observed in the groups were expressed as mean and standard deviation and compared using the Student t test. P values <0.05 were considered statistically significant.

## RESULTS

The study population had a mean age of 48±13.63 years and consisted of 81% of women. Approximately 59% were classified as ASA I; surgery had an average duration of 77±28.70 min, and from all patients two (2%) had infection, one in group A and one in group B. No differences were found between the groups in terms of mean age (p>0.05) or the time of surgery (p>0.05). No associations were observed between the ASA and the use of antibiotic prophylaxis (p>0.05) or gender (p>0.05). They also found no associations between the use of antibiotic prophylaxis and the occurrence of infections postoperatively (p>0.05).

## DISCUSSION

The use of prophylactic antibiotics in surgery still causes controversy among surgeons. One should take into account that their misuse increases the rate of infection and involves unnecessary cost. There is evidence that there is no indication of antibiotic use in clean and potentially contaminated operations, where the risk of surgical site infection is up to 5%. However, in daily practice is not uniform, and the use of prophylactic antibiotics is common in these situations.

According to the Center for Disease Control - USA most postsurgical infections are acquired during the surgical procedure, and good technique is crucial to its prevention. In addition, the center published consensus on surgical site infection prevention in which they emphasized the main points of prevention; among them; the administration of antibiotics must be taken intravenously as a single dose or while the operation is being performed, or at most for a few more hours after skin closure, not exceeding 24 h^25^.

The literature, in most studies, demonstrates that there is no need for antibiotic prophylaxis in laparoscopic cholecystectomy. Ruangsin et al.^18^ studying 299 patients in a prospective randomized study showed no significant benefit in reducing the incidence of postoperative infection. Similarly other authors referred the same results^16^ ^,^ ^21^ ^,^ ^22^ ^,^ ^26^ ^,^ ^29^.

Graham^7^ auditing 111 surgeons in Great Britain and Ireland involving over 7,000 laparoscopic cholecystectomies pointed out that more than 20,000 doses of antibiotics were used unnecessarily. A similar study conducted by Jaafar^10^ involving 13,911 patients also found no benefit in prophylactic antibiotic administration, as well as Kacelnik^11^ in Norway.

Meta-analysis^22^ ^,^ ^27^ studying antibiotic prophylaxis in laparoscopic cholecystectomy in randomized trials with a significant number of patients (n=1937) also demonstrated that prophylactic antibiotics are not required for elective laparoscopic cholecystectomy in low risk patients^11^ ^,^ ^12^.

 Moreover Matsui et al.^13^ in randomized controlled clinical study of 1038 patients who underwent laparoscopic cholecystectomy observed a significant decrease in the incidence of infectious complications in the group receiving antibiotic prophylaxis. They also reported decreased hospitalization costs due to lower rate of infection. However, we should make some considerations regarding this article. The time of postoperative hospital stay of 3-5 days is much higher than the expected 24-48 h; they included distance infections in the results - urinary tract, respiratory, prostatitis, colitis and fever - in the first 24-48 h as evidence of infection running away from the goal of antibiotic prophylaxis, which is the surgical site. Another finding conflicting in this paper is the prophylatic dosage performed in a total of three doses of 1 g of cefazolin: first, immediately before the skin incision and the second and third 12 h and 24 h, against the international guidelines that limit antibiotic dose at induction of anesthesia and repeated or not during operation in relationship to the extension of the operation. And finally, when comparing this Matsui^13^ paper with existent meta-analyzes^22,27^ is observed less scientific relevance in the Japanese study.

The NNIS system is considered standard for predicting risk of surgical site infection. It should be considered as precipitating factor for infection in surgical site, in addition to the comorbidities of the patient, contamination potential of the procedure, completion time in larger procedure over percentile 75 and laparoscopic cholecystectomy longer than 120 min^25^.

In this series the patients were similar considering NNIS score, demonstrating the homogeneity of the groups as low risk for surgical site infections. The incidence of infection was small, 2% in each group, with no difference between making or not antibiotic prophylaxis. The literature has also shown that antibiotic prophylaxis does not have significant role in the prevention of surgical site infection in laparoscopic cholecystectomy and increases the costs of the procedure, so discouraging its routine use.

## CONCLUSION

The use of antibiotic prophylaxis in laparoscopic cholecystectomy have no benefit in reducing the incidence of surgical site infection.
